# Canine sperm vitrification with nonpermeable cryoprotectants and coconut water extender

**DOI:** 10.1590/1984-3143-AR2023-0004

**Published:** 2023-06-23

**Authors:** Anton Antonov, Boyana Ivanova

**Affiliations:** 1 Department of Obstetrics, Reproduction and Reproductive Disorders, Faculty of Veterinary Medicine, Trakia University, Stara Zagora, Bulgaria

**Keywords:** dog sperm, vitrification, lecithin, sucrose, coconut water

## Abstract

This study was aimed to assess the efficiency of coconut water extender with addition of soy lecithin and sucrose as nonpermeable cryoprotectants for canine semen vitrification, using a simple method that yields a high survival rate of spermatozoa for clinical use. Twelve ejaculates from 12 adult normozoospermic dogs were collected separately by digital manipulation and only the second semen fraction was used in this study. After evaluation of volume, concentration, viability, total and progressive motility, velocity parameters and morphology, semen was diluted with a coconut water extender (50% (v/v(volume per volume)) coconut water, 25% (v/v) distilled water and 25% (v/v) 5% anhydrous monosodium citrate solution) with addition of soy lecithin and fructose at 1% and 0.25M sucrose until final concentration of 100x10^6^ spermatozoa/ml. After equilibration at 5ºC for 60 minutes, semen was vitrified by “direct dropping method” into liquid nitrogen in spheres with a volume of 30 μl. After a week of storage the spheres were devitrified as three of them were dropped into 0.5 mL of CaniPlus AI medium (Minitüb, Germany), which was previously warmed in a water bath at 42ºC for 2 minutes and evaluated about the above mentioned parameters. It was found that vitrification resulted in a lower percentage of viable sperms, normal morphology, total and progressive motilities (p<0.05), but most of velocity parameters (VCL, VSL, VAP, LIN, ALH and BCF) did not differ (p>0.05) compared to fresh semen samples. In conclusion, our results demonstrate that vitrification with coconut water extender with addition of 1% soy lecithin and 0.25M sucrose as cryoprotectants, has an excellent potential for routine canine sperm cryopreservation.

## Introduction

In recent years there has been a demand on research for different methods for canine semen preservation (Eilts, 2005). There have been many trials based on type of preservation, various component of extenders, equilibration and warming procedures. Cryopreservation of spermatozoa is a method for assisted reproductive biotechnology, useful for extending their lifespan and viability, which increases reproductive capacity of male organisms (Gharajelar et al., 2016). Two types of sperm cryopreservation are developed until now - conventional and vitrification. Conventional cryopreservation uses slow-gradual freezing method and has moderately poor post-thawed semen quality (Baiee et al., 2020). Vitrification on the other hand uses an ultra-rapid freezing method for solidifying liquid into glassy state by direct immersion into liquid nitrogen (LN_2_) avoiding ice crystal formation (Isachenko et al., 2004; Amirat-Briand et al., 2010; Magnotti et al., 2018). The method is widely used for embryo, oocyte or tissue storage (Isachenko et al., 2004; Rosato and Iaffaldano, 2013) and during last decade it has been successfully developed in some animal species as an option for sperm cryopreservation, however in dogs there are less investigations until now (Sánchez et al., 2011; Kim et al., 2012; Gharajelar et al., 2016; Caturla-Sánchez et al., 2018; Pipan et al., 2020; Galarza et al., 2021; Antonov and Ivanova, 2022). As a novel method, sperm vitrification protocols still require improvement and standardization for increasing post thaw sperm survival.

Different semen extenders were discovered and developed in order to protect spermatozoa from different harmful factors (Bustani and Baiee, 2021) and choosing the right one is an important part of semen processing (Peterson et al., 2007; Ogbu et al., 2014). Commercial extenders for canine semen cryopreservation, which consist of chemical combinations are available and they differ in their ingredients and complexity. There are variable natural extracts and infusions with cryopreserving properties useful as alternative sources of semen extenders for preserving animal sperms (Sansone et al., 2000). Most of them have high antioxidant capacity, which plays protective role on spermatozoa from oxidative damage during cryopreservation process (Tezcan et al., 2009). One of the natural buffer solutions, suitable as biological ingredient of semen extender is the coconut water (Cardoso et al., 2003, 2005, 2006; Gunawan et al., 2016; Puja et al., 2018). It is isotonic, not toxic, cheap, effective, and simple to be used (Cardoso et al., 2003), with high antioxidant properties (Cardoso et al., 2003, 2006; Silva and Bamunuarachchi, 2009; Mantena et al., 2003), containing a lot of phytohormones (Nunes and Sales, 1993), sugars, vitamins, electrolytes and amino acids (Yong et al., 2009).

During the semen cryopreservation process the addition of cryoprotectants is mandatory to minimalize cryodamage of the spermatozoa (Enciso et al., 2006). Two groups of cryoprotectors are available - permeable and nonpermeable. Most often glycerol is added to the extenders as permeable cryoprotectant, which prevents intracellular ice crystals formation, but it has proven to be toxic for the cells (Curry, 2000; Holt, 2000). Different combinations of carbohydrates (sucrose, lactose and trehalose) and proteins (bovine serum albumin, milk, lecithin or egg yolk) are useful as nonpermeable cryoprotectors (England, 2000; Isachenko et al., 2004), which prevent water precipitation and formation of intracellular or extracellular ice crystals by greatly increasing viscosity of the extender (Isachenko et al., 2011). Egg yolk has been widely used in mammalian sperm cryopreservation to protect sperm from initial cold shock (Layek et al., 2016; Abdel-Aziz Swelum et al., 2019), however in recent years there is a trend of using animal free ingredients in extenders in order to avoid restrictions in worldwide semen transport.

There are limited data about the potential of using coconut water extender with addition of nonpermeable cryoprotectors to preserve important physiological parameters of canine sperms during ultra-rapid cryopreservation (Antonov and Ivanova, 2022). Current research was aimed in optimizing the vitirification and post warm recovery of canine spermatozoa using coconut water extender with addition of 1% soy lecithin and 0.25M sucrose as cryoprotectants.

## Methods

### Experimental animals and initial semen quality

Twelve privately owned, clinically healthy male English bulldogs, aged between 3 – 6 years, were included in this study. They were presented at the University Veterinary Hospital of the Faculty of Veterinary medicine, Trakia university, Stara Zagora, Bulgaria. Semen samples from these dogs were previously conventionally cryopreserved and found to be with good cryotolerance. In the present experiment the animals did not undergo any harm or suffering and did not receive any experimental treatment, so ethical approval from the Local Animal Ethics Committee was not required. Dog owners were informed about the procedures and signed a written consent before semen collection. Inclusion criteria were that the ejaculates should have ≥70% motile spermatozoa and ≥70% morphologically normal spermatozoa.

### Semen collection and evaluation

Semen was collected separately by digital manipulation into a pre-warmed sterile plastic vial. The procedure was performed by the same operator to eliminate variation due to different collection technique and in the presence of a teaser bitch to provide stimulation. Only the second ejaculation fraction was used in the study and immediately after collection the vial was transferred to the laboratory for the preliminary analysis. Semen was evaluated for volume, concentration, vitality, kinematic parameters and morphology.

Pre-warmed graduated glass pipette was used to measure the semen volume.

Sperm concentration and velocity were analyzed by CASA System Sperm Class Analyser (SCA) (Microptic, S.L., Barcelona, Spain) with software analytical module Motility and concentration, using a Makler counting chamber with volume of 10 µl samples. The CASA parameters were adjusted to accommodate canine semen according to protocols already in place. Examination was performed on a minimum of 30 optic fields. The assessed CASA parameters were: sperm concentration (x10^6^/ml), total motility (TM, %), progressive motility (PM, %), VCL (curvilinear velocity, µm/s), VAP (average path velocity, µm/s), VSL (straight line velocity, µm/s), LIN (linearity, %), STR (straightness rate, %), ALH (lateral head displacement amplitude, µm) and BCF (beat cross frequency, Hz).

Sperm morphology was evaluated by Sperm Class Analyser (SCA) (Microptic, S.L., Barcelona, Spain) with software analytical module Morphology. Smeared 10 µl semen samples were prepared on a clear glass slide and stained with SpermBlue (Microptic, Spain) for 2 minutes. The slide was analyzed by SCA with a minimum of 50 optic fields.

The sperm viability was assessed by smearing on a slide a mixture of 5 µl semen sample and 5 µl eosin-nigrosine stain. A minimum of 200 sperm cells were counted under a light microscope and oil immersion, magnified by 400x. Spermatozoa stained pink or red were identified as no vital, and those unstained remaining white - as vital.

### Extender preparation and semen dilution

After the initial processing, the second semen fraction was diluted with a green coconut water based extender until final concentration of 100 x 10^6^/ml. The extender consisted of 50% (v/v) water from green coconut, 25% (v/v) distilled water and 25% (v/v) 5% anhydrous monosodium citrate solution with addition of soy lecithin and fructose at 1% (v/v) and 0.25 M sucrose. The extended semen was then equilibrated at 5˚C for 1 hour.

### Vitrification and thawing

The vitrification technique was performed according the description of Shah et al. (2019) for human sperm. Ten aliquots of 30 µl of all sperm samples were dropped with micropipette from a 10 cm height upon a stainless steel strainer which was previously placed into a styrofoam box and submerged in liquid nitrogen (LN_2_). After solidification the sperm pellets were transferred into pre-cooled cryotubes and placed in liquid nitrogen for a week.

**Figure 1:**
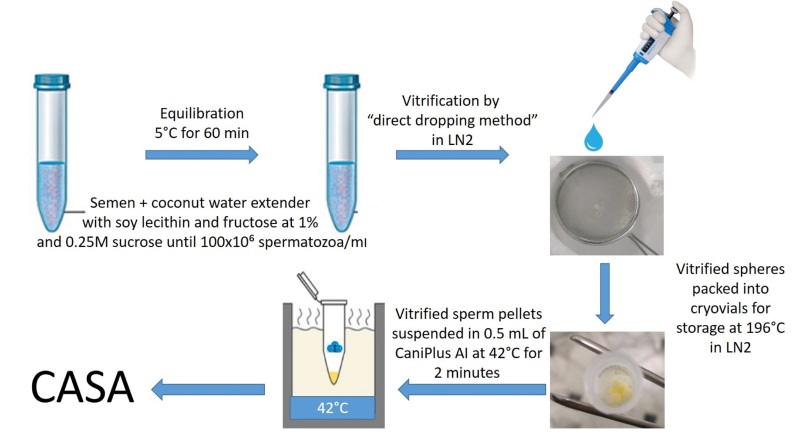
Different steps of canine spermatozoa vitrification process.

The thawing process consisted of adding three sperm pellets of each sperm sample to 0.5 ml of CaniPlus AI medium (Minitub, Germany) that has been pre-warmed in a water bath at 42˚C for 2 minutes and then transferred at 37˚C. After warming the sperm parameters were immediately evaluated as described above. All the steps for semen cryopreservation are presented on Figure 1.

### Statistical analysis

The results were processed by statistical program Statistica version 7.0 (Stat-Soft., 1984-2000 Inc., Tulsa, OK, USA). All data is presented as mean ± standard deviation (SD) and was analyzed using ANOVA for repeated measures and compared using the Tukey’s test. Value for p<0.05 was considered significant.

## Results

The volume of the second semen fraction was 0.95±0.27 mL and the concentration - 973±175.04×10^6^ spermatozoa/mL. The other evaluated parameters and the effect of vitrification on canine semen using coconut water extender with addition of soy lecithin and sucrose are presented on Table 1.

**Table 1:** Parameters of fresh and devitrified canine semen samples (n = 12) using coconut water extender. Data are expressed as mean ± SD.

	Viability, %	Total motility, %	Progressive motility, %	VCL, µm/s	VSL, µm/s	VAP, µm/s	LIN, %	STR, %	ALH, µm	BCF, Hz	Normal morphology, %
Fresh semen	94.45±1.59	87.76±1.94	52.11±2.89	191.1±26.60	129.2±16.60	146.3± 15.10	68.50±8.36	88.93±4.31	5.11±0.71	25.1±3.60	84.08±5.56
Vitrified semen	60.93±3.28 a	53.23±4.56 a	45.11±1.15 a	179.3±26.9	122.5±15.6	143.9±17.2	67.68±6.75	85.38±4.63 a	4.89±0.87	22.5±4.1	70.71±5.12 a

The values in a row marked with a superscript differ at P < 0.05.

Fresh semen samples showed significantly higher (p<0.05) viability than those which were vitrified. Similar tendency was observed in total motility and progressive motility. After vitrification there were also changes in most of sperm velocity parameters, however they were not significantly different (p>0.05) than the fresh samples before cryopreservation.

The evaluation of sperm morphology showed significant variations (p<0.05) between the fresh and vitrified samples. The major alterations found after vitrification were detached head, coiled and bent tail.

## Discussion

In order to increase the reproductive capacity of stud dogs, in recent years a lot of scientific research has been done, focused on canine semen cryopreservation. Conventional freezing methods usually result in high percent sperm mortality and morphological damage (Baiee et al., 2020), but they are still prefered for cryopreservation of canine semen (Kim et al., 2012). On the other hand, vitrification by “direct dropping method” into liquid nitrogen has its own unique characteristics and the result is solidification without formation of hexagonal (big, lethal) intracellular crystals by extreme increase in viscosity during cooling (Merino et al., 2011).

Scientists working in the field of reproduction are interested in the possible potential and health benefits of different phytochemicals and the synergistic effects of their ingredients as complex compared to the single purified active fractions (Seeram et al., 2005). Semen cryopreservation is a useful method in order to preserve the valuable genetic constitution of stud dogs, but usually results in cryodamage induced by freezing and thawing, which could be decreased by using proper ingredients or suitable cryoprotectants into the semen extender. Our choice to use coconut water based extender relays on the cheap, easy to find ingredient, which is also an excellent antioxidant and is in accordance with the “green trend” of recent years. In our opinion, coconut water could safely be used as a part of semen extender, because it is sterile and there is no need to add antibiotics, which usually have some hazardous effect on sperm cells. An evidence for this hypothesis is that the quality of preserved canine semen after devitrification in the present study exceeded any previously reported results (Sánchez et al., 2011; Kim et al., 2012; Gharajelar et al., 2016; Caturla-Sánchez et al., 2018; Pipan et al., 2020; Galarza et al., 2021).

According to Gharajelar et al. (2016) egg yolk is a common part of semen diluents with protective effect on spermatozoa against cold shock during freezing and thawing, which also acts as an energy source and protectant at the level of the cell membrane (Sánchez et al., 2011). In the scientific literature the best canine sperm viability and total motility reported after vitrification were with TRIS based extender (Pipan et al., 2020) and previous study of our team using coconut water extender (Antonov and Ivanova, 2022). In both investigations the extenders contained 1% soy lecithin and 0.25M sucrose as cryoprotectants. In this study, the improved recovery rates after vitrification compared to previously reported of our team (Antonov and Ivanova, 2022) could be due to the 42˚C temperature used for warming as compared to 37˚C in the previous studies of sperm vitrification. In order to provide best sperm survival, the freezing rate must be added by a suitable thawing temperature regimen, which could be even more critical than the cooling rate in vitrification (Mazur and Seki, 2011). In previous investigations of dog sperm vitrification, warming protocol has largely been ignored or unintentionally missed, but as it is already known, both are highly correlated. During thawing, the osmotic balance is reversed, rehydration occurs and the lipid protein configuration of the membrane is restored similarly as the events are induced during freezing (Simons, 2018). Our results confirm the beneficial effect of thawing temperature on the examined parameters, which is in agreement with Fizer et al. (1993) that warming also has a very critical role in sperm survival as cooling does, because sperm survival and damage depends on the intermediate zone of temperature between -10 to -60°C and they have to traverse through it twice during a cryopreservation protocol. Further research on enhancement in warming procedures should be conducted and investigated in order to improve the protocols for canine sperm vitrification.

Motility is one of the most important features of a fertile spermatozoa (Partyka et al., 2012). In previously reported results for conventional freezing, wide variability is observed, with studies reporting sperm motility ranging between 33% (Peña et al., 1998) and 70% (Ström et al., 1997). Concannon and Battista (1989) suggest that 30–50% sperm motility in frozen semen is considered acceptable and according to Linde-Forsberg and Forsberg (1989) motility above 50% is ideal for artificial insemination with canine frozen semen. Our result showed that despite the significant difference in total motility between the fresh and vitrified sample, the mean average percent of sperm motility after devitrification is above mentioned levels and thus can be successfully used for artificial insemination.

In our research we found that there were no significant differences (p>0.05) between the velocity parameters (VAP, VSL, VCL) and BCF of fresh and vitrified samples, which is in agreement with previously reported results of our team (Antonov and Ivanova, 2022). Their evaluation is the most useful method for comparing semen from fertile and infertile dogs (Domosławska et al., 2013), because of their importace for the progression of sperms into cervical mucus and zona pellucida penetration of oocytes (Verstegen et al., 2002). According to the reported results, sperm vitrification yields a high survival rate of fertile canine spermatozoa after warming.

Our study demonstrate that canine sperm vitrification in a coconut water extender with addition of 1% soy lecithin and 0.25M sucrose could be successful for routine clinical use as alternative to conventional cryopreservation. This ultra-rapid freezing method is much faster, simpler and cheaper method, avoiding high spermatozoa mortality rates observed in conventional freezing. Additionally, we believe that coconut water could successfully replace some of the expensive chemical ingredients of semen extenders and has a positive impact on the environment. Therefore, further research on fertility studies should be conducted and investigated to detect true measure of successful dog sperm vitrification with coconut water extender.

## Conclusion

In conclusion, when coconut water extender with addition of 1% soy lecithin and 0.25M sucrose as a cryoprotectants were used, vitrification affects the quality of canine spermatozoa, but could provide quality results near the conventional freezing methods and could be useful as their alternative in routine canine sperm cryopreservation.
